# A Column-Generation and Branch-and-Cut Approach to the Bandwidth-Packing Problem

**DOI:** 10.6028/jres.111.015

**Published:** 2006-04-01

**Authors:** Christine Villa, Karla Hoffman

**Affiliations:** George Mason University Mail Stop 4A6 Systems Engineering and Operations Research Department 4400 University Drive, Fairfax, VA 22030

**Keywords:** capacitated networks, column generation, combinatorial optimization, cutting planes, facet-defining cuts, heuristics, integer programming, network planning, telecommunications networks, zero-one optimization

## Abstract

The telecommunications problem of assigning calls with point to point demand to a capacitated network where each call can be assigned to at most one path has been called the Bandwidth-Packing Problem. For a given network, with specified arc costs and arc capacities, one wishes to route calls (defined by a starting and ending point) through the network to maximize the profit from the calls routed. Each such call is single path routed and not all calls will be routed. We propose a branch-and-cut methodology coupled with column generation to optimally solve such problems. We examine the alternative approaches in the literature and explain how this new method takes the best of all components of methods suggested previously. The method we suggest is new in that it includes a linear programming-based heuristic for obtaining good lower bounds, uses lifted minimal covers that take into account special-ordered set constraints, and dynamically choose among three alternative branching strategies. In addition, whenever a new column is generated, it is lifted into all existing cuts. We also discuss the need to generate all tied optimal linear optimization solutions if one wishes to assure that the solution obtained is optimal. Our computational results provide solutions to problems previously unsolvable.

## 1. Introduction

The bandwidth-packing problem is a combinatorial optimization problem arising from telecommunication networks where demand exceeds capacity and where point-to-point calls with varying bandwidths are routed on this network such that each call uses a single path. These problems occur within ATM technology and when video data is routed. It belongs to the broad class of multi-commodity flow problems that are commonly associated with communication, computer, transportation, and distribution network applications. Calls between pairs of nodes define the commodities, and links connecting nodes represent transmission lines in the telecomm network. Given a set of calls with their revenue and bandwidth requirements and an undirected network with its fixed arc/link capacities and costs, the problem is to assign calls from a request table to paths such that network capacities are not violated and profit is maximized.

When one can split a call among a variety of paths, then the problem can be solved by linear programming techniques. However, there are many telecommunications problems (as well as applications outside telecommunications, see Barnhart et al. [2000]) where the commodity must be routed on a single path. This additional requirement makes the problem NP hard, and few papers address methodology to tackle this problem. We present a technique that has become popular recently for difficult, large 0–1 problems: that of performing both column-generation and row-generation within a tree-search. We also include a heuristic to improve bounds on the problem.

### 1.1 Problem Formulation

The bandwidth-packing problem (BWP) can be formulated as a 0–1 integer-programming problem over a network defined by nodes *i* and arcs or links *l* that connect such nodes. A path consists of a route for a given call *i* through a collection of links that connects a source node *s_i_* to terminal node *t_i_*. The objective function is to maximize profit and the decisions to be made are whether to route a given call and if routed, to determine which of the multiple paths to pick to route the call.

Let the 0–1 decision variable *x_i j_* indicate whether call *i* is routed on path *j*. Then the objective is to maximize profit from assigning calls to paths, such that calls are assigned at most once and link capacities are not violated. The profit of path *j* is defined as the revenue for call *i*, *r_i_*, minus the total bandwidth cost of using the links in path *j* (i.e., the profit of path *j* associated with call *i* is equal to: $r_{i}-d_{i}\sum_{l=1}^{m}\delta_{l_{j}}c_{l}$) where $\delta_{l_{j}}$ is an indicator that equals one whenever link *l* is used in path *j*, *d_i_* is the bandwith requirement (demand) of call *i*, and *c_l_* is the per unit bandwidth cost of using link *l*. We assume that there are *n* calls and *m* links in the network.

Then with the following notational definitions:

Call *i*: *r_i_*: revenue for call *i*
*d_i_*: bandwidth requirement (demand) of call *i*
*s_i_*, *t_i_*: source node and terminal node of call *i*
*P_i_*: the set of paths for call *i*
Link *l*: capacity of link *l*, in bandwidth
*c_i_*: unit cost of bandwidth on link *l*.
Variable: *x_ij_*: *l* if call *i* is assigned to path *j*; 0 otherwise.

We obtain the integer linear optimization problem:

$$\max\sum_{i-1,\cdots ,n}\sum_{j\in P_{i}}\left[r_{i}-d_{i}\sum_{i-1,\cdots ,m}\delta_{l_{j}}c_{l}\right]x_{ij}$$

*Subject to*:

$$\begin{array}{ll} \sum_{j\in S_{i}}x_{ij}\leq 1 & \underset{routing\;constraints\;\left(SOS\right)}{\forall i=1,\ldots ,n\in \text{calls --}}\\ \sum_{i=1,\cdots ,n}\sum_{j\in P_{i}}\delta_{l_{j}}d_{i}x_{i}{}_{j}\leq b_{l} & \underset{capacity\;constraints\;\left(\text{KNAPSACKS}\right)}{\forall 1=1,\ldots ,m\in \text{links --}}\\ x_{i}{{}_{j}}_{\in }\{0,1\} & \forall i=1,\ldots n,j\in P_{i} \end{array}$$

The first set of constraints insures that each call is either routed or not, grouping the paths by call into non-overlapping special-order sets. The second set of constraints is a collection of knapsack constraints that insure no link capacities are violated. All variables for a given call share a single coefficient (the bandwidth requirement for the call) for each knapsack constraint. (Note that *x_ij_* = 1 is implied by the routing constraints, so explicitly setting the upper bound to one is not necessary.)

### 1.2 A Small Example

**Fig. 1:**
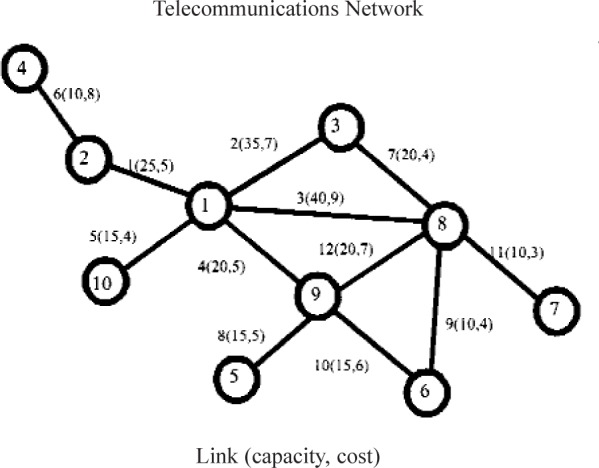
ASample Telecommunications Network with Node Numbers, Link Numbers, Link Capacities and Link Costs Identified. Asample call table is also given.

We illustrate the problem structure with the following problem (Fig. 1).

**Table:** Call Table

Call	si/ti	di	ri
1	1/3	10	420
2	1/4	7	380
3	1/10	6	400
4	1/	6	390
5	2/7	5	500
6	2/6	5	490
7	2/5	7	400
8	3/10	2	150
9	3/4	4	450
10	3/5	8	500
11	4/6	6	850
12	6/3	3	200
13	7/10	5	370
14	8/2	6	500
15	8/10	5	340
16	8/5	2	120
17	9/2	6	460
18	9/3	8	450
19	10/6	5	360
20	10/2	5	170

si = source node
di = bandwidth demand
ti = terminal node
r = revenue

This problem (Fig. 1) then translates to the following A-matrix structure (Table 1) where there are a collection of special-ordered set constraints and a set of knapsack constraints, where in each knapsack, all paths (variables) associated with the call have the same coefficient. The reason for this commonality is that, for a given call, each of the paths uses the same amount of bandwidth. In table 1 we note that paths P1 through P4 relate to call 1, paths P5 through P10 relate to Call 18 and paths P11 through P14 relate to call 14.

In this paper, we test our approach using the standard data sets used in earlier work. All problems have the same basic two data sets: a call table and a link table. The call table lists the call’s source and destination nodes, its demand, and its revenue in units of bandwidth. The link table specifies for each link its adjoining nodes, its bandwidth capacity, and the cost per unit of bandwidth using the link. We show the paths related to only three calls (call 1: (node 1 to node 3), call 18 (9 to 3) and call 14 (8 to 2)) of this problem to illustrate the structure of the A-matrix.

Since there is no requirement to route a call in this formulation, only profitable paths need to be considered. While the number of nodes in the network typically will not be large (<40 nodes in our test problems), the number of profitable paths increases quickly with the number of links.

Parker and Ryan (1994) showed the bandwidth-packing problem is NP-Hard since it contains a number of 0–1 knapsack constraints as part of the entire constraint set.

Even for small networks, the number of possible paths generated for each call through pure enumeration can be enormous. Incurring this upfront cost of generating all such paths when most will not have any possibility of being in the optimal solution is unnecessary. Instead, one prefers to generate columns only as needed, that is, only when they will improve the lp-solution. This approach is known as “column generation” and requires a “pricing” algorithm that either generates such a column or proves that no such column exists. Since the linear programming solution serves as an upper bound on the solution value, one wants this upper bound to be as close to the true integer solution as possible. One can tighten the linear programming relaxation by applying cutting planes that more closely approximate the convex hull of the feasible integer points.

Given a good upper bound on the solution value, one must also have a good lower bound on the solution for a bounding procedure such as branch-and-bound to work efficiently. We employ an lp-based heuristic for obtaining good lower bounds on the problem. This paper will present a solution methodology that incorporates all of these techniques and uses a dynamic branching strategy based on setting many variables simultaneously to zero, to improve the overall search. We begin by summarizing the prior research on this problem.

### 1.3 Previous Work on This Problem

Initial work on the bandwidth-packing problem focused on heuristic methods. Cox et al. (1991) described the problem for dynamic call routing where the bandwidth-packing problem is solved as a subproblem. They did not envision the number of nodes in the network to be large (30 to 50 nodes) but the number of calls could be as high as 100. Even in sparse networks, the number of different paths possible for a single call can be quite large. Also, the number of possible routes increases with the density of the network. Cox et al. used a genetic algorithm and permutation based approach to path assignment. Each potential solution is represented as a permutation of an ordering of the calls and is evaluated by assigning the shortest path available after previous calls in the ordering are routed. Anderson et al. (1993) also used a permutation approach but within a tabu search. Laguna and Glover (1993) developed a non-permutation tabu search algorithm, where potential solutions are evaluations of changes in path assignment for a given call. Gavish and Altinkemer (1990) and Fischer et al. (1994) used Lagrangian Relaxation to obtain good feasible solutions to special cases of the bandwidth-packing problem. Amiri and Barkhi (1999) consider a multi-hour bandwidth-packing problem and also use Lagrangian Relaxation to obtain heuristic solutions to their problems. A number of recent papers have examined how to assign calls to links to minimize the queueing delay when arrivals are stochastic: see Rolland et al. (1999) and Amiri et al. (1999).

Since our paper is concerned with proving optimality to difficult bandwidth-packing problems, we spend more time examining the prior work on exact methods. Parker and Ryan (1994) describe a column-generation branch-and-bound procedure (also known as branch and price).

The problem was formulated as an integer programming problem by applying column generation based on the linear programming relaxation imbedded in a branch and bound procedure. Their branching strategy chooses a certain fractional path for a given call. They then create *k* + 1 branches. On *k* of these branches, they stipulate that the call cannot use arc/link *j* (*j* = 1…*k*). The *k* + 1st leaf requires that the call not use any of the arcs/links. They dynamically set the optimality tolerance and solve all but two instances of their problem set within 95 % of optimality. They prove optimality to only two of these fourteen problems, however. Parker and Ryan do not use cutting planes.

Park et al. (1996) embed cutting planes based on lifted minimal covers (see Padberg 1975) within column generation to create the *lp*-relaxation, and incorporate these cuts into a branch-and-cut procedure. Cuts were not generated within the tree unless the branch-and-price routines failed to prove optimality within a reasonable period of time. Then, cutting plane routines were also employed in the branching tree. The authors employed a traditional *x_i j_* = 0/1 branching strategy; however, for comparison they implemented a simplified version of Parker and Ryan’s approach and showed that the use of cutting planes substantially improves solution times for either branching rule. We note that the test-set of problems used by Park et al. were randomly generated problems and appear to be much easier to solve than those presented in the Parker and Ryan or those in the Glover and Laguna papers.

Recently, two other papers use a branch-cut-and-price algorithm for the bandwidth-packing problem. Barnhart et al. (2000) again use lifted minimal covers within a branch-cut-and-price approach. Their branching strategy is different from that of either Park et al. or Parker and Ryan. They identify a call that is split between two paths and then identify the node such that prior to that node the two paths are identical, and call this sub-path *s*. They call this node the *divergence node*, labeled *d*, and the two arcs that cause the divergence *a*_1_ and *a*_2_. They partition all arcs leading from divergent node *d* into two disjoint partitions such that one set A_1_ contains *a*_1_ and the other set A_2_ contains *a*_2_. On one branch all paths that contain subpath *s* and any arc in A_1_ are set to zero and on the other branch all paths that contain subpath *s* and any arc in A_2_ are set to zero. Thus, this branching approach has two properties: (1) it is easy to generate new paths for a call without changing the pricing of new columns and (2) many variables (paths) might be fixed to zero on a single branch. They use a depth-first tree search in order to obtain good feasible solutions early in the search. They discovered that on many branches the objective function changed little because of the symmetry in the problem. They therefore incorporated lifted cover inequalities and perform this lifting based on the algorithm of Gu et al. (1995). We note that the authors do not use the special-ordered sets to strengthen these cuts. The success of these authors was impetus for us to see if providing stronger cuts and providing a heuristic might improve upon their results.

Finally, Geffard (2001) also uses a branch-cut-and-price approach. Here, the minimal covers are not lifted but a heuristic is used to obtain good bounds. The authors branch on arcs for a specific call, similar to one of the three branching strategies that are employed in this paper. We will discuss this branching strategy later. Their testing showed that using a heuristic enhanced the performance of their code significantly.

### 1.4 Contributions of This Paper

In our view, the contributions of this paper include:

1. the incorporation of much stronger cuts into a branch-cut-and-price code for the bandwidth-packing problem. These lifted minimal covers with special-ordered sets are easily incorporated into the pricing algorithm and, whenever a new path is generated for a given call that uses a link not previously used by *any* paths for that call, the variable (path) is lifted into all cuts associated with that link. This lifting calculation is inexpensive to perform.
2. the inclusion of a dynamic branching strategy that does not require significant changes to the pricing algorithm.
3. the inclusion of an *lp*-based heuristic that quickly finds very good solutions and is capable of being used throughout the tree.
4. the solution of previously unsolved problems in the standard test-set of bandwidth-packing problems.
5. a small example that illustrates the need for generating all optimal solutions to the linear programming problems on each branch.

### 1.5 Outline

The remainder of this paper is organized as follows. Section 2 describes each sub-algorithm of the branch-cut-and-price code. Section 3 provides computational results on two published sets 13 of test problems. Section 4 provides conclusions and avenues for future research.

## 2. Solution Methodology

Since pricing and cutting are complementary procedures for tightening an *lp*-relaxation, our method generates both columns and cuts throughout a branch and bound tree. Because there are too many possible columns to handle efficiently and most of them will have their associated variable equal to zero in an optimal solution, a more efficient approach than enumeration is to start with only a small subset of the feasible columns and then add others as needed. This process is called “pricing” and uses reduced-cost information to add columns *only* when they can improve the linear programming solution value.

Likewise, as the above discussion has highlighted, adding cutting planes based on the polyhedral structure of the 0–1 polyhedron is a very efficient way of tightening the linear programming approximation to the integer-programming problem.

In addition, we add an *lp*-based bounding heuristic to obtain a good *ip*-lower bound early and we attempt to update this bound whenever columns and cuts are generated. Having both a good upper bound and a good lower bound allows variables to be fixed based on the “gap” (i.e. the difference between the *lp*-solution and the best-known feasible solution).

The basic algorithm begins by solving the *lp*-relaxation of the BWP problem with only a subset of paths S*_i_* – P*_i_* per call. Then, for each call, we generate columns (paths) based on dual prices. When no additional columns will improve the *lp*-solution, we begin generating cutting planes for each link constraint coupled with the entire set of special-ordered set (SOS) constraints. These cutting planes are considerably stronger than those generated when the SOS constraints are ignored. We continue cycling through cut generation and column generation until we can find no additional cuts or columns to improve the *lp*-solution. At this point, we perform tree search where at each branch of the tree, we again employ *both* column and cut generation. In addition, we employ an *lp*-based heuristic within the branching tree. Figure 1 presents the overall algorithm. We present each of the sub-algorithms below.

### 2.1 Solving the First Linear Program

Our approach to generating columns is similar to that of prior authors. For the first linear program, we use the cost on the links to determine at least one shortest path for each call. Where the solution to the shortest path problem is not unique, we generate *all* shortest paths. For data sets where the link costs were nonzero, there was usually only one or two optimal paths per call. However, for data sets where the link costs were all zero, generating all shortest paths generated many per call, so we only added up to 5 paths (columns) per call in the initial formulation of the linear program. However, prior to branching, we added *all* paths of a call that had a reduced cost of zero. This approach is different from Parker and Ryan who use only one shortest path for the problem, or Park et al. who use a k-shortest path algorithm (but in most of their paper report test results for k set equal to 1). Given at least one path for each call, we solve the first *lp*. We now present the general pricing algorithm which is performed after every *lp* call.

### 2.2 Generating Columns

By *lp*-duality theory, the *lp*-solution is optimal if and only if the dual solution is feasible. If any of the dual constraints are not satisfied for any call, then there exists a column or columns that would have been in the basis had they been in the *lp*-relaxation. We use the same column generation scheme as Parker and Ryan. It is briefly described here. Let *y_i_* denote the dual variables corresponding to the routing constraints, *z_l_* and the dual variables corresponding to the capacity constraints *y_i_*, and let *S_i_* denote the current subset of paths in the *lp*-relaxation. Call this problem LP^S^.

$$\max\sum_{i-1,\cdots ,n}\sum_{j\in P_{i}}\left[r_{i}-d_{i}\sum_{i-1,\cdots ,m}\delta_{l_{j}}c_{l}\right]x_{ij}$$

*Subject to*:

$$\begin{array}{ll} y_{i}:\;\sum_{j\in S_{i}}x_{ij}\leq 1 & \underset{routing\;constraints\;\left(SOS\right)}{\forall i=1,\ldots ,n\in \text{calls --}}\\ z_{l}:\;\sum_{i=1,\cdots ,n}\sum_{j\in P_{i}}\delta_{l_{j}}d_{i}x_{i}{}_{j}\leq b_{l} & \underset{capacity\;constraints\;\left(\text{KNAPSACKS}\right)}{\forall 1=1,\ldots ,m\in \text{links --}}\\ x_{i}{{}_{j}}_{\in }\{0,1\} & \forall i=1,\ldots n,j\in S_{i} \end{array}$$

The solution to the *lp*-relaxation using a subset of paths *S*, (*LP^S^*) is optimal for the *lp*-relaxation *LP^P^* if and only if the dual solution returned by the simplex method is feasible for the dual of *LP^P^*. The dual constraint associated with variable *x_i j_* (corresponding to call *i* using path *j*) is:

$$\begin{array}{l} y_{i}+d_{i}\sum_{l=1,\cdots ,m}\delta_{l_{j}}z_{l}\geq r_{i}-d_{i}\sum_{l=1,\cdots ,m}\delta_{l_{j}}c_{l}\\ \text{or}\;d_{i}\left[\sum_{l=1,\cdots ,m}\delta_{l_{j}}(z_{l}+c_{l})\right]\geq r_{i}-y_{i} \end{array}$$

where δ*_lj_* = 1 if link *l* is in path *j*; 0 otherwise.

We must determine whether a path *j* for call *i* is in the path set *P_i_*, but not in our generated paths *S_i_*, could improve the *LP^S^* solution. The column-generation subproblem associated with call *i* is therefore:

$$\begin{array}{l} \min\;d_{i}\left[\sum_{l=,\cdots ,m}\delta_{lj}(z_{l}^{*}+c_{l})\right]\\ subject\;to:j\in P_{i} \end{array}$$

where $y_{i}^{*}$ and $z_{l}^{*}$ are the dual values returned by the simplex method in the current *lp*-relaxation. Thus, given a network with link weights $d_{i}(z_{l}^{*}+c_{l})$, one finds the shortest path from the source node to the destination node of call *i*. If the solution to this shortest path problem has path lengths less than $r_{i}-y_{i}^{*}$, the current *lp*-solution is not optimal. Path *j* should be added to the problem and the linear program re-solved. Since link weights are non-negative, a modified Dijkstra’s (see Dial et al. 1979) algorithm can be used to find the shortest path(s) for the column-generation subproblem. We also note that by generating *all* shortest paths for *all* calls, we are likely to limit the number of times that the pricing routine will need to be called.

Our formulation of the column-generation subproblem differs from Parker and Ryan’s in that we have a third set of dual variables corresponding to the dual prices on the cut constraints. Since our cuts are based on capacity constraints, and since capacity constraints relate to individual links, the cuts we generate add an additional cost to each link in the shortest path. It is therefore straightforward to augment the shortest path problem with these dual prices.

Specifically, if *K* is the number of cuts so far generated, where *π_ik_* is the coefficient for call *i* in cut *k*, and *π*_0_*_k_* is the right-hand-side value of the cut constraint, then we denote the cut in the following form:

$$w_{k}:\;\sum_{i=1,\cdots ,n}\sum_{j\in S_{i}\subset P_{i}}\delta_{l_{i}}\pi_{ik}x_{ij}\leq \pi_{0k}\;\forall k\in K$$

where *w_k_* will denote the dual variable corresponding to cut-constraint *k*.

Then the column-generation subproblem associated with call *i* is:

$$\begin{array}{l} \min\;d_{i}\sum_{l=1,\cdots ,m}\left[\delta_{l_{j}}(Z_{l}^{*}+c_{l})\right]+\sum_{k=1}^{K}\delta_{l_{j}}\pi_{ik_{l}}w_{k}^{*}\\ subject\;to:j\in P_{i} \end{array}$$

From this formulation of the shortest path problem, we see that the weights on each arc/link become $d_{i}(z_{l}^{*}+c_{l})+\sum_{k=1}^{K}\pi_{ik_{l}}w_{k}^{*}$. As will be seen in the next section, the cuts we generate relate to the capacity of any link and are based on minimal covers for the capacity constraints (knapsacks) in the problem. Once a cut is generated for a call that uses that link, the coefficient $\pi_{ik_{l}}$ in the cover is included in that link’s cost in the shortest path problem. If the solution to this shortest path problem is less than $r_{i}-y_{i}^{*}$, then the current *lp*-solution is not optimal and we have found another path to add to the problem.

### 2.3 Generating Cuts

Once the *lp*-relaxation is dual feasible, cutting plane constraints are generated and added to the *lp*-relaxation to better define the feasible integer solution space. When incorporated into a branch- and-cut procedure, these additional constraints reduce the solution space for the linear programming relaxation, while not cutting off any portion of the solution space that contains a feasible integer point. We used facial cuts similar to those described in Hoffman and Padberg (1991) and Crowder et al. (1983). We also exploit the fact that all paths for a call that use the same link have the same coefficient in the capacity constraint. This fact coupled with the fact that only one path can be chosen assures that the lifting coefficient on *every* such path is equal. Thus, one can find the lifting coefficient for *every* path of a given call by performing only one optimization! This result is a direct application of the theorem of Johnson and Padberg (1981) that provides a strong lifting procedure when one considers knapsacks with disjoint special-ordered sets.

Minimal-covers are generated based on the link capacity constraints and the set of special-ordered-set constraints:

$$\begin{array}{ll} \sum_{i=1,\cdots ,n}\sum_{j\in S_{i}}\delta_{l_{j}}d_{i}x_{ij}\leq b_{l} & for\;some\;link\;constraint\;l\\ \sum_{j\in S_{i}}x_{ij}\leq 1 & \forall i=1..n \end{array}$$

In a manner similar to that described in Hoffman and Padberg (1991), we project out variables at both zero and at one. We then solve the knapsack problem with special-ordered sets *only* over the fractional variables. We then *sequentially* lift back any fractional variables not in the minimal cover, then all variables projected out at one, and finally those at zero.

Specifically, we consider some link *l* and let $x_{ij}^{*}$ be the optimal solution to the current *lp*-relaxation. Define the following sets over the variables *x_i j_* that use link *l*: $Q_{1}=\{x_{ij}^{*}|x_{ij}^{*}=1\}$, $Q_{0}=\{x_{ij}^{*}|x_{ij}^{*}=0\}$, and $Q_{F}=\{x_{ij}^{*}|0<x_{ij}^{*}<1\}$. Also, let the set *T_i_* be the set of calls for which there is at least one $x_{ij}^{*}$ fractional. We then solve the following minimal-cover problem:

$$\xi =\min\{\sum_{i\in T_{i}}(1-\sum_{ij\in Q_{F}}x_{ij}^{*})z_{i}|\sum_{i\in T_{i}}d_{i}z_{i}>b_{l}-\sum_{i\in Q_{1}}d_{i},z_{i}\in \{0,1\}\}$$

If *ξ* < 1, then a valid inequality has been found which cuts off $x_{ij}^{*}$. We note that since *all* paths for this call that use this link require the same bandwidth, each such path has the identical coefficient value in the link-capacity constraint. Thus, if *any* path for this call is part of the minimal cover, then all paths for that call that use this link will also be part of the cover with the same cut (cover) coefficient. We can therefore perform the “lifting” of new paths that use a specific link into an existing minimal-cover constraint with virtually no additional computational effort.

In cut generation, we use strong lifting to tighten the problem. Once a minimal cover is found, we lift back any fractional variables that were not in the original cover, then lift back the variables that were projected out at one (see Wolsey, 1975 for details) and then lift back the variables at zero (see Padberg, 1975). Since these lifting procedures are *sequential*, different lifting orderings will generate different facets. We vary the order and generate up to five lifted minimal-cover inequalities for each link-capacity constraint.

We also lift newly generated paths (new columns in our *lp*-formulation) into the existing cuts because the special structure of this problem allows us to lift *all* paths for a call simultaneously. We can do this since the coefficient in the knapsack constraint is the same for all such paths. Thus, from the Johnson and Padberg result, we know that all such variables will have the same lifting coefficient. We keep track of whether a call has *any* paths that use a specific link. If, at some future time, we generate a new path for a call that uses a link previously unused for that call, we lift this new path into *all* cuts that are associated with that link. Thus, even as the dimension of the problem expands, we are able to maintain cuts based on the facial structure of this higher-dimensional polyhedron.

### 2.4 A *lp*-Based Heuristic

A good feasible solution provides a lower bound to the problem. Parker and Ryan documented the value of having a good lower bound early. When they fed their algorithm the best known solution obtained by Anderson et al., problems were solved faster and/or the solution quality improved in most cases. We therefore created a *lp*-based heuristic which we call after every round of column and cut generation at the top of the tree. Within the tree, we call the heuristic before exiting the branch. Establishing a lower bound early on has several advantages. Variables can be fixed based on their reduced cost and the gap between the *lp*-optimal and *ip*-optimal solution-values. Fathoming rules can be applied earlier to prune nodes from the enumeration tree. Also, if the user requires a solution to within a specified tolerance, the entire algorithm can be terminated as soon as the lower and upper bounds differ by less than this tolerance.

The heuristic uses the current *lp*-relaxation in a procedure similar to the “Dive and Fix” algorithm described by Wolsey (1998). First, the heuristic fixes variables currently at 0 in the current *lp*-solution to 0 and variables at 1 to 1. Then it sets all fractional variables with *lp*-solution values greater than 0.7 to 1, or if none > 0.7, it selects the fractional variable closest to 1, fixes it to 1, and re-solves the linear program. One of three cases will result: (1) the solution is integer—stop and save this solution if it is better than the previous best *ip*-solution; (2) the *lp* is infeasible—stop; or (3) the linear program is feasible but the solution is not integer—repeat. We repeat, fixing variables and re-solving until the set of unfixed fractional variables is no more than 12. Given that it is quite fast to completely enumerate the 2^12^ possibilities, we enumerate and choose the best of all feasible solutions.

At the top of the tree before initiating branching, we do a rigorous search for good *ip*-solutions easily available from the current *lp*-relaxation. We begin by fixing all variables at 0 to 0 and all variables at 1 to 1. Then, each fractional variable greater than 0.5 is, in turn, is set to 1. This *lp* is solved and then the *lp* heuristic described above is performed. Since the order in which fractional variables are set to 1 matters, repeating the *lp*-heuristic for each “candidate” variable (x^*^_ij_ > 0.5) searches for *ip*-solutions from the most likely set of variables.

In our experiments the heuristic often found the optimal integer solution at the top of the tree or early within the branching tree. The rigorous heuristic at the top of the tree significantly improved the quality of the *ip*-solution in most cases. For comparison, we sent the *lp*-relaxations at the top of the tree to ILOG CPLEX 8.0 (2002) to solve the integer program. CPLEX often visited several hundred nodes before finding the optimal solution. Also, because we were not dependent on a branching rule to find good feasible solutions, we chose to use the node having the best (i.e., largest) objective function value; we call this a best-node search of the branching tree. Both Park et al. and Barnhart et al. use depth-first search in order to find feasible solutions.

### 2.5 Branching

We begin branching when no additional columns can be found that improve the *lp*-solution and we cannot find any cuts that cut off the current fractional value.

Our branching strategy is rather different from those used in the past. Park, Kang, and Park used traditional branching in their branch and bound algorithm, where each node in the tree opens two new branches; one forcing the selected *x_i j_* to 1 and the other to 0. When *x_i j_* is forced to 1, this is a very strong branch since all other paths for that call are set to zero. However, the other side of the branch, when *x_i j_* is set to 0, is very weak. This branching scheme also impacted their pricing scheme. Often, they would generate a column that they would have to discard because it violated the branching constraints.

As described earlier, Barnhart et al., chose a branching strategy that tried to alter the objective value on both branches of the current node, and also assured that their shortest path calculations were not harmed by this branching scheme. We use a different branching rule, which also maintains these positive characteristics.

Since we already have a good *ip*-lower bound, we search the tree using the best node and use a hybrid branching strategy to select the branching variable and rule. The hybrid strategy uses one of three branching schemes based on which one indicates that it will alter the *lp*-bound on both new branches.

There are three possible branching rules:

1. Choose a call *i*, and on one side, force this call into the solution and on the other side specify that this call will not be routed. ( $\sum_{j\in S_{i}}x_{ij}=1$, on one side; and $\sum_{j\in S_{i}}x_{ij}=0$ side of the branch for some call *i*.)
2. Choose a call *i*, and choose a capacitated link *l* based on its dual price. On one branch, we specify that this call *must* use link *l* and on the other side, we specify that this call *cannot* use link *l.*( $\sum_{j\in S_{i},\partial_{l=1}}x_{ij}=1$.on one side of the branch, and $\sum_{j\in S_{i},\partial_{l=1}}x_{ij}=1$ on the other side for some call *i and link l.*)
3. Choose a path *ij* for call *i* that is fractional and branch on that path. *(x_i j_* = 0 on one side of the branch, and *x_i j_* = 1 on the other for some call *i* and path *j*.)

We have listed these branching rules in the order that they are applied in the algorithm. First, we check if there is slack on any of the routing constraints. If so, we branch on the call with the most slack. On one side of the branch, we change the constraint from an inequality to an equation and on the other side we force all paths for that call to be zero. This forces the linear program to decide whether or not to route the call.

When all routing constraints are tight, we identify the fractional variable *x_i j_* closest to 0.5 and its most capacitated link *l*. We then specify that either the call uses this link or it does not. This is equivalent to saying $\sum {}_{j\in S_{i},\delta_{l_{j}}=l}x_{ij}=0$ on one branch and $\sum {}_{j\in S_{i},\delta_{l_{j}}\neq l}x_{ij}=0$ on the other. Thus, instead of only fixing one variable, we fix a set of variables on each side of the branching tree. To prevent paths that use this link (on the branch that does not allow such paths), we set the link weight for link *l* to infinity. Similarly, if we wish to generate more paths that use this link, we can generate a shortest path from the source of call *i* to link *l*, and then a shortest path from link *l* to the destination of call *i*.

Finally, if the selected variable *x_i j_* is the *only* path for call *i* that uses link *l*, then we do the traditional branching on variable *x_i j_*.

## 3. Importance of Generating All Optimal or Assuring That Every Branch Has a Feasible Linear Programming Solution

We now discuss why one needs to generate *all* optimal solutions to the linear programming problem within the branch and bound tree. All rules for column-generation procedures that we are aware of require only that one obtain an optimal solution at the top of the branching tree and that one use the normal fathoming strategies within the tree: fathom if (a) the linear programming problem is infeasible, (b) the linear programming problem produces a feasible integer solution, or (c) the linear programming solution provides an answer worse (or at least not better than) the best known integer solution.

We present a simple example that shows that using the standard rules (those currently published in virtually all) column generation papers is insufficient to guarantee optimality. Specifically, the literature states that one need only show that there is no column that *improves* the objective function (i.e., that the bound at the top of the tree is a proper bound). Once one has obtained this bound, one can then branch on a fractional variable and, if one then gets a proper bound at each node in the branching tree, when the tree is fathomed, one has obtained the optimal solution to the overall problem. We present a simple (seven variables, three constraints) set-partitioning problem that—when following these standard rules for branching and fathoming—does not generate the columns needed to find the optimal solution. Thus, the standard algorithm would conclude that there are *no* integer feasible solutions to a problem that has *multiple* optimal integer solutions.

All rules for column generation procedures that we are aware do not discuss this problem. In Barnhart et al. (1998) they mention that the *initial* restricted master problem must have a feasible *lp*-relaxation to ensure that proper dual information is passed to the pricing problem. However, they do not indicate that one needs a similar requirement *throughout* the tree. Thus, it is implied that once one has obtained an optimal linear-programming solution at the top of the branching tree, one can use the normal fathoming strategies within the tree: fathom if (a) the linear programming problem is infeasible, (b) the linear programming problem produces a feasible integer solution, or (c) the linear programming solution provides an answer worse (or at least not better than) the best known integer solution. We note that adding artificial variables that ensure that a feasible solution to the LP relaxation at the top of the tree exists is not sufficient to not cut off optimal integer solutions throughout the tree. Our example will illustrate this point.

We present a simple example that shows that one needs to be very careful about fathoming rules for a column-generation approach. We present a simple (seven variables, three constraints) setpartitioning problem that shows that a column-generation phase must take place at each node of the branching tree—even when the linear programming relaxation obtains an infeasible or integer solution.

### 3.1 Example

Consider a set-partitioning problem with three constraints and where the possible columns are: (1,0,0), (0,1,0),(0,0,1),(1,0,1),(1,1,0),(0,1,1), and (1,1,1). Let the objective value for each column be equal to the sum of the ones in that column,. The respective objective function values are therefore (1, 1, 1, 2, 2, 2, 3). Now all feasible solutions to this problem have objective value = 3. Assume that one begins the column-generation process with the following three columns: (1,1,0), (1,0,1) and (0,1,1). The linear program solution for this three column problem is z^*^ = 3 with a solution vector of (1/2,1/2,1/2). Since the master problem has a feasible *lp*-relaxation, one has proper dual information at the top of the tree and there is no need to generate an artificial column. Now, within the column generation process, we find that all other columns have reduced cost of zero, so no column can improve the objective function value. We stop and decide to branch without generating any new columns. We therefore choose to fix one of these three variables (columns) to either one or zero and “branch.” On each side of the branching tree, the linear optimization has no feasible solution and we conclude (incorrectly) that the problem has no feasible points! This is clearly false.

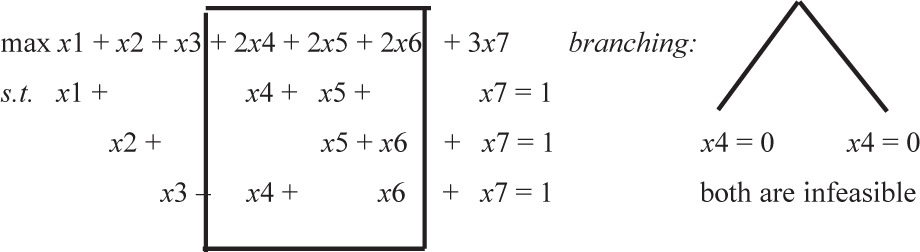


This simple problem highlights that the current literature has not completely specified what is needed to guarantee that important columns have not been ignored in a column-generation scheme. Instead of using the “normal” branch-and-bound fathoming rules, one must go through a column-generation phase at each node. In the case where solving the linear program provides an integer linear programming solution, one can use the dual prices from this solution to start the column-generation phase. In this case, the node will be fathomed only if, after column-generation, the solution remains integer. In the case where one obtains an infeasible solution, one needs to add artificial variables that make up a feasible solution (with the requisite high cost) in order to obtain the dual prices needed to begin the column-generation phase. If, after this column-generation phase, the solution remains infeasible, the node be fathomed.

## 4. Computational Results

The software developed to test these ideas was written in C and runs on a PC in a Linux environment. CPLEX 8.0 is used to solve the *lp*s using the simplex method with all preprocessing turned off. All problems were run on Dell OptimPlax PC with Red Hat Linux 8.0 operating system. ILOG CPLEX 8.0 (2002) Software Components library for Linux PC was used for solving all linear programming problems. We used none of the ILOG CPLEX integer programming components—thus, all branching rules, cutting planes, pre-processing and heuristic routines were written in C by the authors of this paper.

For our computational experiments, we used a set of 10 problems generated by US West and obtained by courtesy of Yuping Qiu. Since this set was also used by Laguna and Glover, and Anderson et al. in their work on the bandwidth-packing problem, we can draw comparisons to previous work. Laguna and Glover ran tests on this set of problems with and without link costs, while Anderson et al. show results only for the problems without link costs. IP1 through IP10 are the original problems and we denote the set without link costs with the letter z (e.g., IP1Z). We also ran tests on 14 problems (labeled DATA1 through DATA14) that were used by Barnhart et al. (2000) in their work. Among the two test bed sets, the problems range in size from 10 to 31 nodes, 15 to 61 links, and 20 to 93 calls. We present pictures of each of these problems in Appendix A.

We present our computational results in the three tables below. Table 1 presents our results. This table provides the number of columns and rows generated at the top of the tree and the linear programming relaxation (ZLP) and integer programming objective function value (ZIP) found at the top. We also indicate our success in solving each of these problems under the columns labeled “At Termination.”

Table 3 shows the benefit of using a *lp*-based heuristic to find a good integer lower bound on the test problems. At the top of the tree before the rigorous *lp*-heuristic was implemented, the best *ip*-solution found was between 88.7% and 100% of the *lp*-solution, with the average for the test sets at 95.5 %. After the rigorous heuristic, the average was increased to 97.5 % (ranging from 94.6 % to 100 %). For test problems with the largest gap (*Z_IP_* less than 95 % of *Z_LP_*), performing the rigorous heuristic reduced the gap by an average of 5.4 percentage points. At the top of the tree, the *ip*-solution found by the heuristic was the optimal solution in 54 % of the problem sets however; it often required considerable branching to prove optimality. In four cases, we terminated the branching at 3000 nodes and could not prove optimality.

In Table 4, we present the prior best known solutions found by other researchers and compare these to the solutions that we obtained.

## 5. Conclusions

In this paper, we have combined the strengths of both column generation and cut generation, exploiting the special structure of the bandwidth-packing problem. It is the first paper to use the strength of special-ordered sets to strengthen minimal-cover cuts within a column-generation setting, and we perform complete lifting. In addition, we have implemented a dynamic branching strategy that works well with our pricing algorithm. This new branching strategy fixes many variables within a single branch by again exploiting the special struc ture of the problem. We have also incorporated a linear-programming based heuristic that seems to find very good solutions to the problem at the top of the tree. This process is especially important when one is not trying to prove optimality, but rather get measurably good integer solutions quickly. This heuristic is usable throughout the tree and can therefore use all of the information that the column generation, cutting planes and branching have provided. The use of this overall algorithm allowed us to find better integer solutions to certain problems than were previously known, allowed us to prove optimality in certain problems, and also showed some of the issues in implementing both column generation and cut generation within a single software package. If dual prices associated with cut constraints are not included in the pricing problem for column generation then columns that were previously generated are likely to be generated again, increasing run time. Finally, we provide a small example that shows that proving optimality to a column-generation code is far more difficult than previously imagined. Specifically, the linear program must not only be solved to proven optimality, but one must also generate *all* alternative solutions to ensure that we do not overlook an optimal column.

## Figures and Tables

**Fig. 2:**
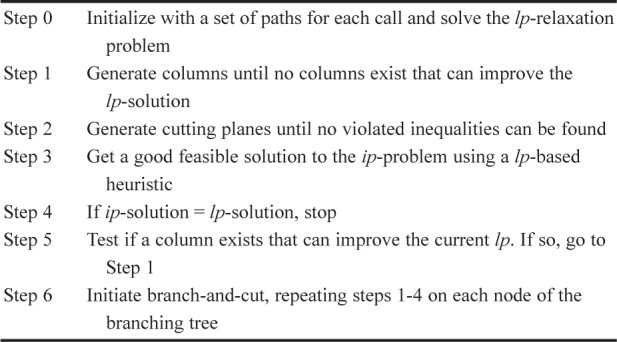
Column Generation/Branch & Cut Algorithm for BWP Problem

**Table 1:** A-Matrix for the sample problem in Fig. 1

Link	Paths														RHS

	P1	P2	P3	P4	P5	P6	P7	P8	P9	P10	P11	P12	P13	P14	
SOS1	1	1	1	1											1
SOS2					1	1	1	1	1	1					1
SOS3											1	1	1	1	1
(1,3)	10					8	8			8	6				35
(1,8)		10					8			8		6			40
(1,2)											6	6	6	6	25
(1,9)			10	10		8							6	6	20
(3,8)		10	10	10	8			8	8		6				20
(9,8)				10	8		8	8					6		20
(9,6)			10						8	8				6	15
(6,8)			10						8	8				6	10

**Table 2:** Computational results for BWP test sets without link costs

Problem	Network/Call input			At the top of the tree				At termination			
	Nodes	Links	Calls	Cols	Rows	*Z_LP_*	*Z_IP_*	*Z_IP_* @ node	Cols	Rows	Branches
DATA1	14	16	35	68	73	6650.0	6580	6580@TOT^a^	69	120	36
DATA2	24	24	68	87	95	7270.0	7270	Solves at TOT			
DATA3	29	61	70	693	349	28738.1	27550	28270@218	1778	2928	3000* b
DATA4	18	29	58	340	153	16826.4	16190	16210@87	488	600	3000*
DATA5	19	25	47	165	84	7790.0	7790	Solves at TOT			
DATA6	27	37	93	285	184	19053.8	18920	18970@426	308	614	862
DATA7	23	29	93	230	195	14109.1	13810	13880@131	307	465	716
DATA8	28	31	41	95	82	8825.0	8770	8770@TOT	100	127	11
DATA9	24	42	87	445	198	21486.8	21360	21360@TOT	700	452	404
DATA10	19	19	41	52	75	7810.0	7640	7640@TOT	52	140	106
DATA11	14	16	23	43	53	6110.0	6110	Solves at TOT			
DATA12	27	36	81	283	160	13290.0	13290	Solves at TOT			
DATA13	29	31	52	141	96	9020.0	9020	Solves at TOT			
DATA14	20	23	46	118	90	8004.4	7900	7900@TOT	136	191	86
IP1Z	10	16	20	133	117	7783.6	7540	7540@TOT	169	617	116
IP2	21	39	20	208	65	2100.0	2100	Solves at TOT			
IP3Z	31	42	50	414	221	14004.3	13570	13710@1284	669	2817	3000*
IP4Z	10	15	20	154	80	3035.7	2925	2955@38	158	548	114
IP5	16	22	20	194	108	2426.2	2295	2395@58	208	252	58
IP6Z	17	26	30	263	130	9319.2	8830	9010@280	267	1002	280
IP7Z	20	31	40	381	244	11235.0	11010	11160@316	428	2957	316
IP8Z	12	18	36	291	130	12810.0	12460	12560@46	372	2915	3000*
IP9Z	12	19	24	200	101	5780.0	5600	5780@4	200	107	4
IP10Z	14	22	28	222	128	1000.9	970	970@TOT	255	813	210

a TOT Stands for “Top of Tree,” *Z_IP_*@node denotes the node at which the integer solution was found.

b * Indicates the test problem was terminated early, before the optimal *ip*-solution was found and/or proven.

**Table 3:** Quality of *ip*-solution at the top of the tree

	*Z_LP_* at Top of Tree	*Z_IP_* before rigorous heuristic	Tolerance	*Z_IP_* after rigorous heuristic	Tolerance
DATA1	6650	6560	98.6 %	**6580**	98.9%
DATA2	7270	**7270**	100 %		
DATA3	28738.1	26170	91.1 %	27550	95.9%
DATA4	16826.4	15540	92.4 %	16190	96.2%
DATA5	7790	**7790**	100 %		
DATA6	19053.8	18920	99.3 %	18920	99.3%
DATA7	14109.1	13810	97.9 %	13810	97.9%
DATA8	8825	**8770**	99.4 %	**8770**	99.4%
DATA9	21486.8	20790	96.8 %	**21360**	99.4%
DATA10	7810	**7640**	97.8 %	**7640**	97.8%
DATA11	6110	**6110**	100 %		
DATA12	13290	13040	98.1 %	**13290**	100%
DATA13	9020	**9020**	100 %		
DATA14	8004.4	**7900**	98.7 %	**7900**	98.7%
IP1Z	7783.6	7530	96.7 %	**7540**	96.9%
IP2	2100	**2100**	100 %		
IP3Z	14004.3	12420	88.7 %	13570	96.9%
IP4Z	3035.7	2770	91.2 %	2925	96.4%
IP5	2426.2	2295	94.6 %	2295	94.6%
IP6Z	9319.2	8830	94.8 %	8830	94.8%
IP7Z	11235	10780	95.9 %	11010	98.0%
IP8Z	12810	12280	95.9 %	12460	97.3%
IP9Z	5780	5500	95.2 %	5600	96.9%
IP10Z	1000.9	920	91.9 %	**970**	96.9%

Note: Bold values are optimal *ip*-solutions found at the top of the tree. However, in several cases of these cases, the solution was not proved optimal until branching was complete.

**Table 4:** Comparison of best known solutions

	Glover and Laguna	Anderson et al.	Parker and Ryan (starting with no *ip* lower bound)	Parker and Ryan (starting with Anderson *ip* lower bound)	Ours
DATA1		6580	6580	6580	6580
DATA2		7270	7270	7270	7270
DATA3^b^		27990	27010	28330	28270
DATA4		16190	16190	16210	16210
DATA5		7790	7790	7790	7790
DATA6		18950	17810	18970	18970
DATA7		13840	13760	13880	13880
DATA8		8770	8770	8770	8770
DATA9		21360	21000	21360	21360
DATA10		7640	7640	7640	7640
DATA11		6110	6110	6110	6110
DATA12^b^		13330* a	13230	13330*	13290
DATA13		9020	8950	9020	9020
DATA14		7900	7900	7900	7900
IP1Z	7540	7540			7540
IP2	2100	2100			2100
IP3Z^c^	13550	13270			13710
IP4Z	2955	2885			2955
IP5^c^	2345	2365			2395
IP6Z	9010	9010			9010
IP7Z	11000	11160			11160
IP8Z^d^	12810	12810			12560
IP9Z	5780	5780			5780
IP10Z	970	970			970

a * Indicates that prior work reported an incorrect optimal solution for problem DATA12. Anderson reported a solution of 13330. Having generated *all* feasible solutions via an enumeration procedure, we believe that no such solution exists. We believe that the best solution to this problem is 13290.

b We also note that within our search-tree, which was truncated at 3000 nodes, the best solution found was 28270 for problem DATA3. We could, not totally enumerate all feasible columns as we did in DATA12 because we ran out of memory after generating *only* the columns for the first call. However, when generating 250000 columns for each call and sending this set of columns to ILOG’s optimization code CPLEX, the code found 36 the solution 28330. thereby confirming that the Parker and Ryan solution of 28330 is feasible and the best solution known to date.

c We note that we have found better solutions to IP3Z and IP5 than had previously been reported.

d For problem IP8Z, we could not find the optimal solution within our 3000-node limit. We did confirm that the solution of 12810 is optimal through a complete enumeration procedure.

**Fig. A1:**
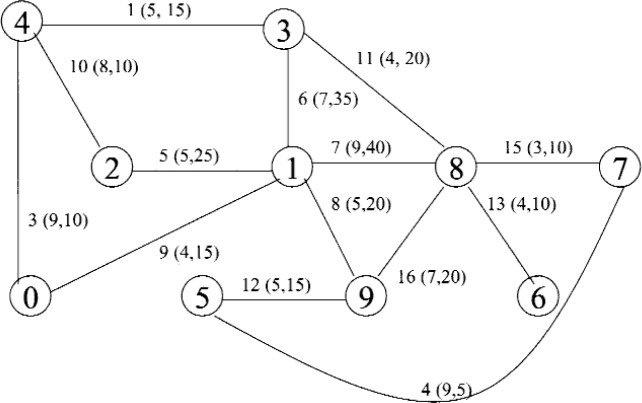
Test Set IP1.

**Fig. A2:**
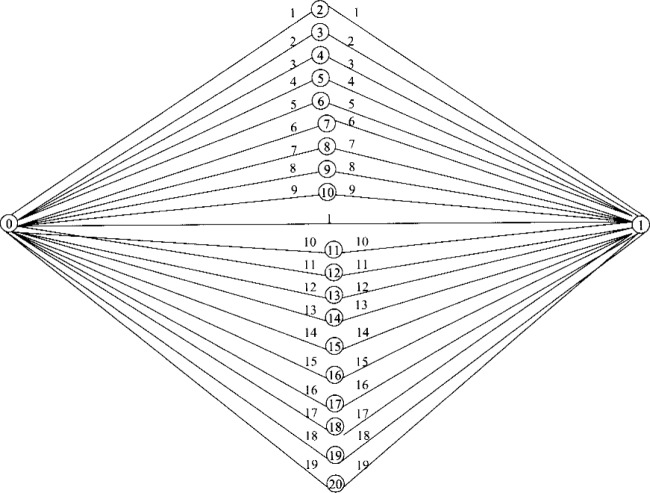
Test Set IP2.

**Fig. A3:**
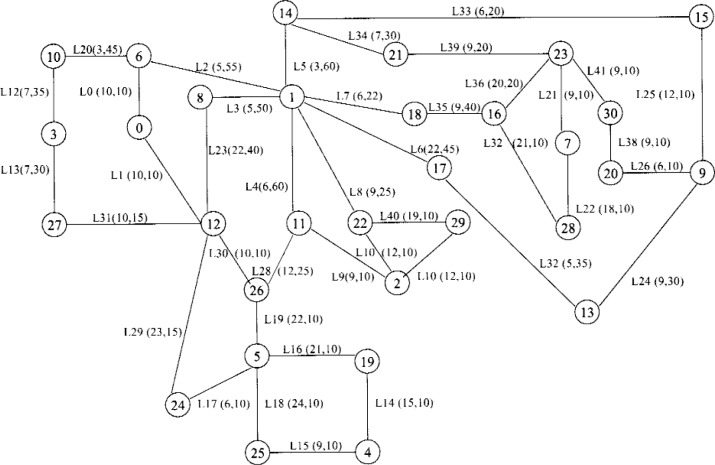
Test Set IP3.

**Fig. A4:**
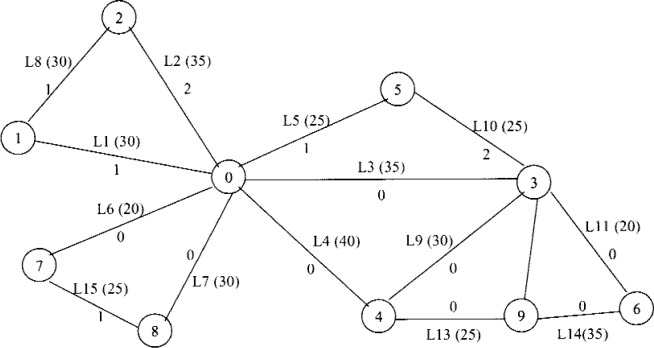
Test Set IP4.

**Fig. A5:**
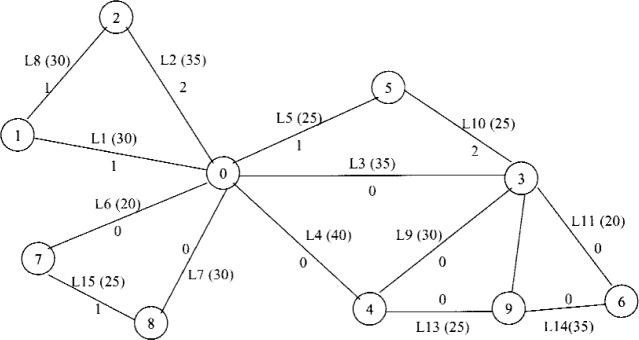
Test Set IP5.

**Fig. A6:**
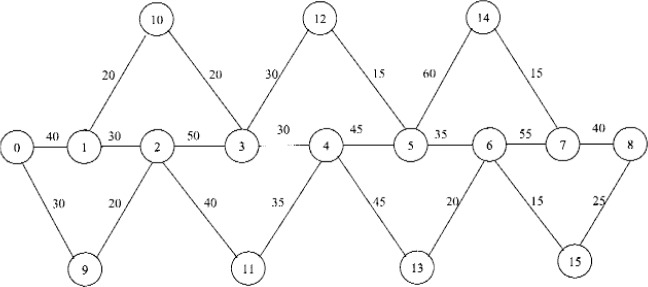
Test Set IP6.

**Fig. A7:**
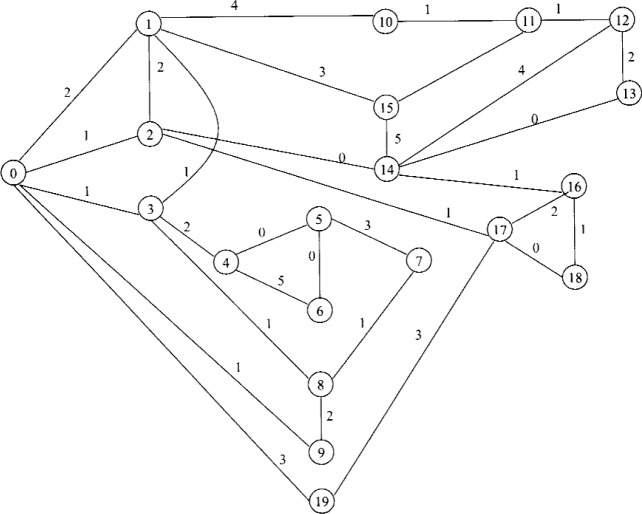
Test Set IP7.

**Fig. A8:**
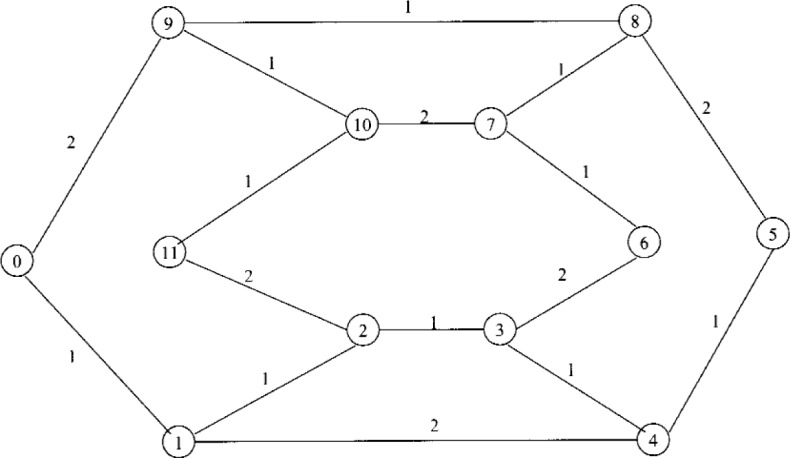
Test Set IP8.

**Fig. A9:**
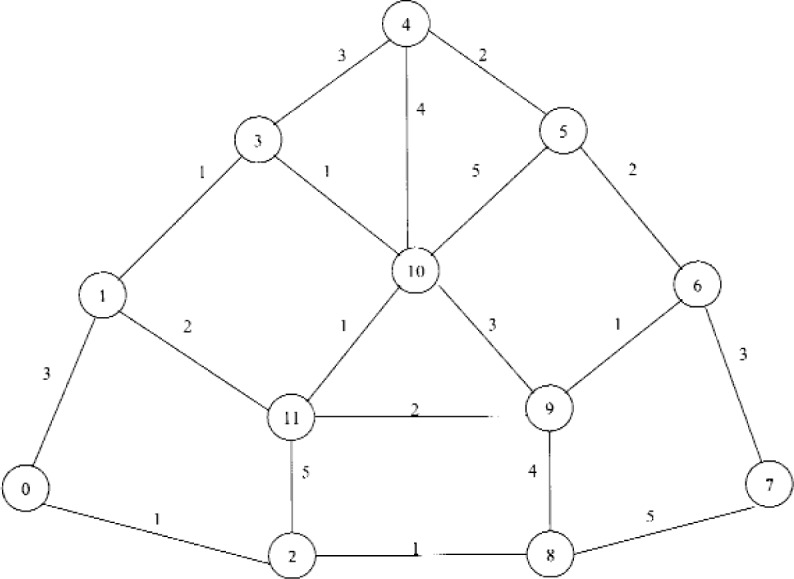
Test Set IP9.

**Fig. A10:**
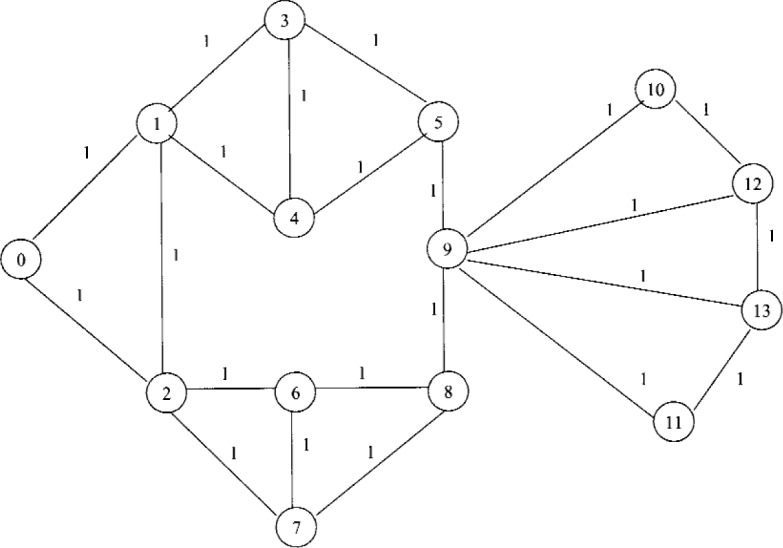
Test Set IP10.

**Fig. A11:**
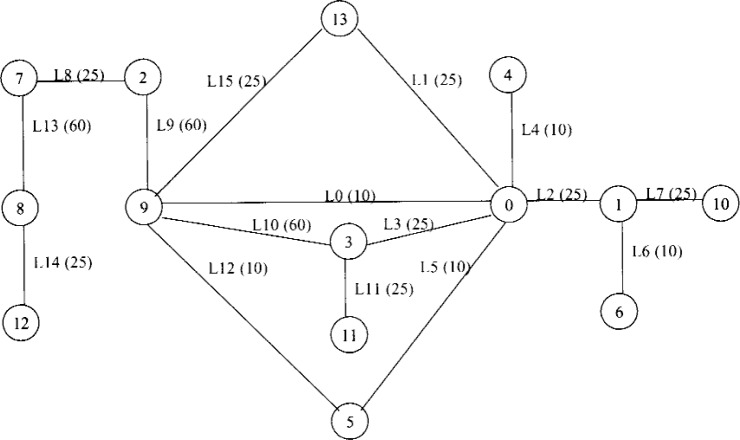
Test Set DATA1.

**Fig. A12:**
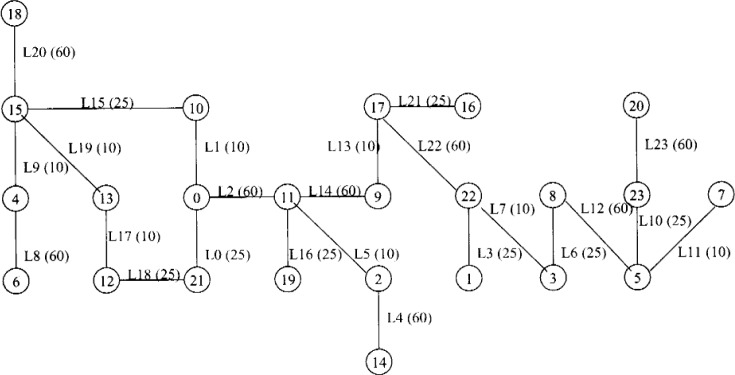
Test Set DATA2.

**Fig. A13:**
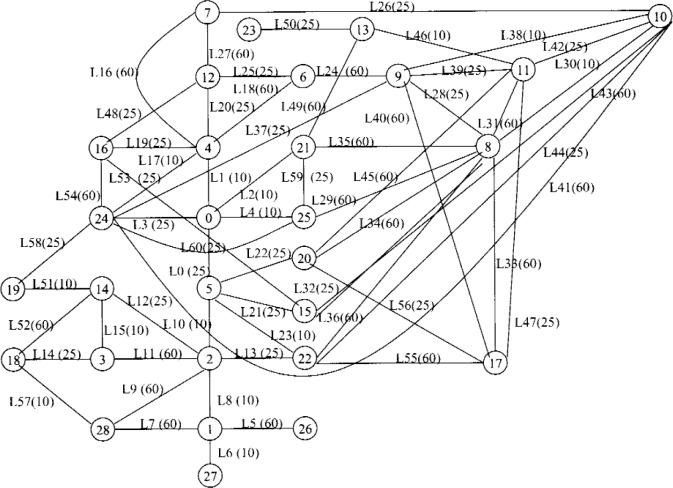
Test Set DATA3.

**Fig. A14:**
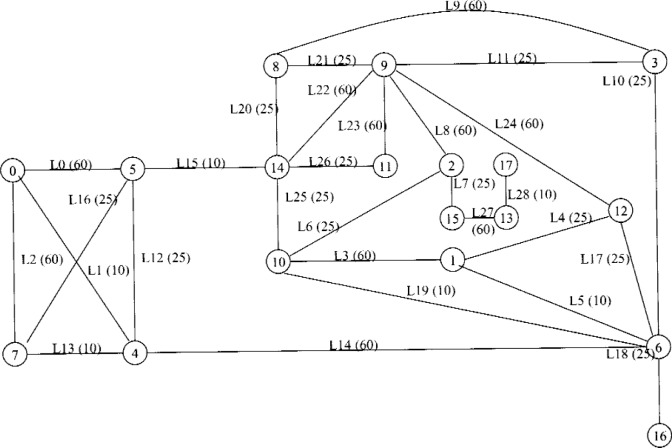
Test Set DATA4.

**Fig. A15:**
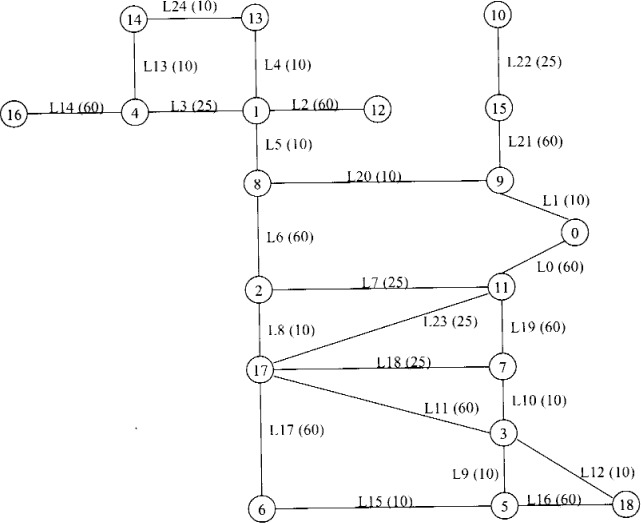
Test Set DATA5.

**Fig. A16:**
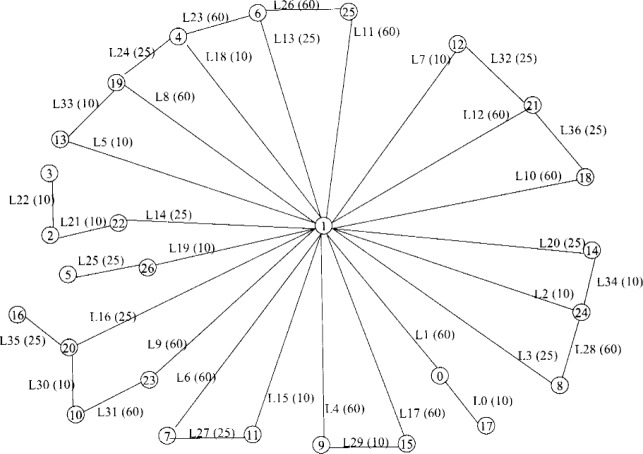
Test Set DATA6.

**Fig. A17:**
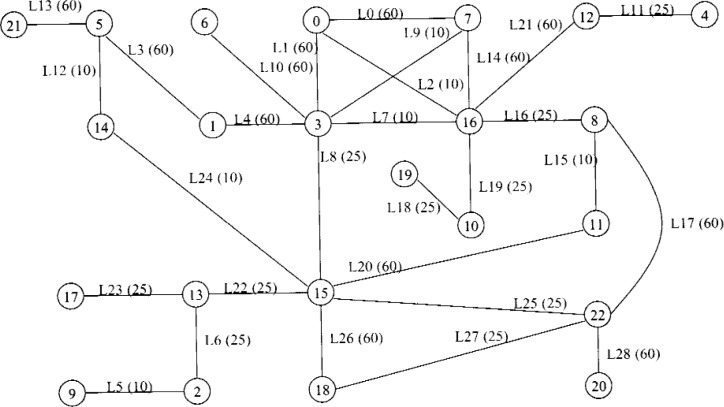
Test Set DATA7.

**Fig. A18:**
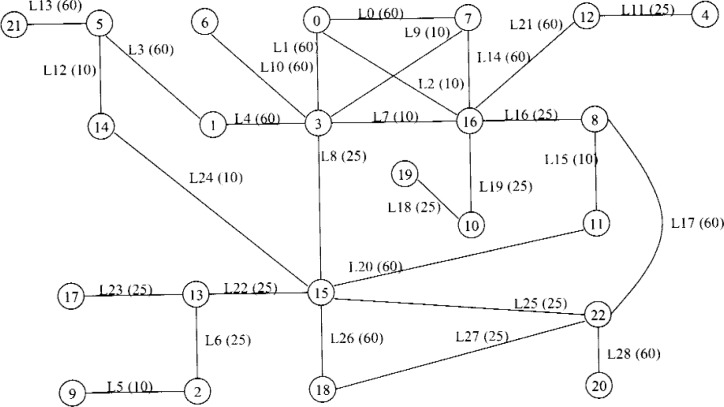
Test Set DATA8.

**Fig. A19:**
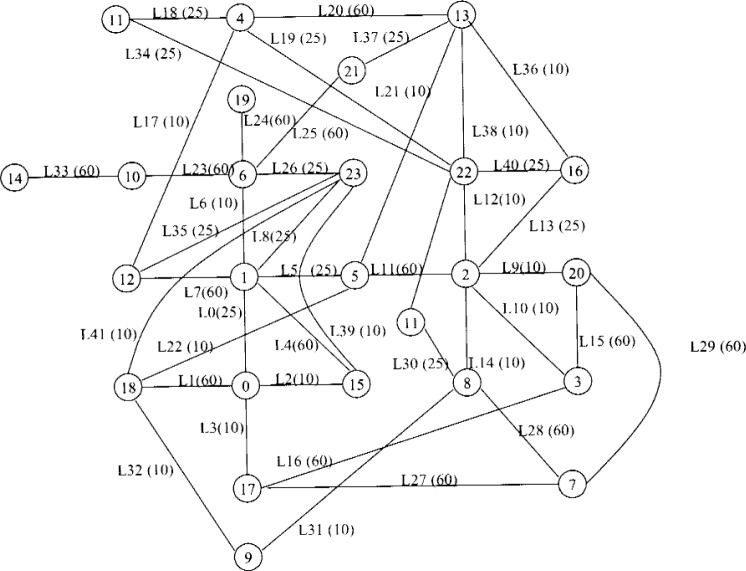
Test Set DATA9.

**Fig. A20:**
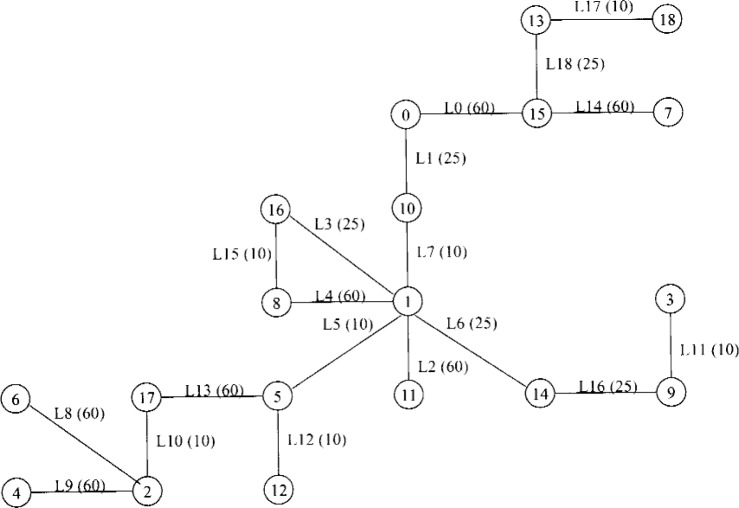
Test Set DATA10.

**Fig. A21:**
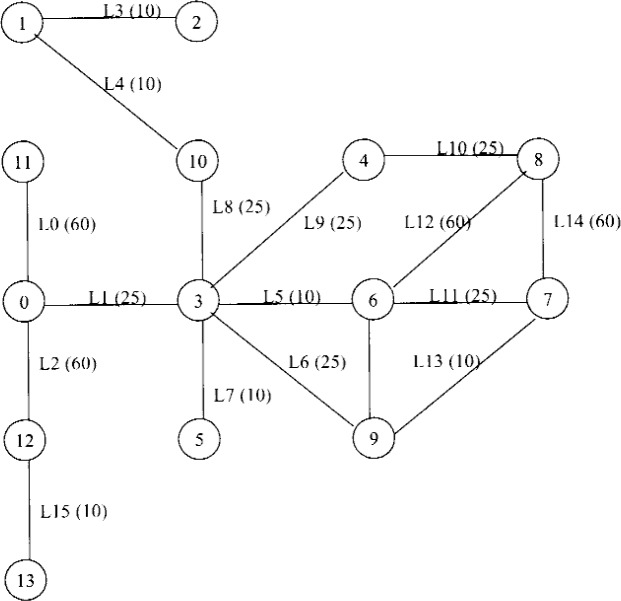
Test Set DATA11.

**Fig. A22:**
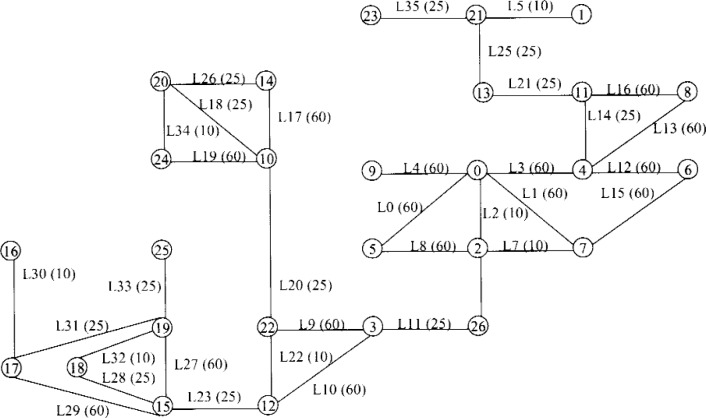
Test Set DATA12.

**Fig. A23:**
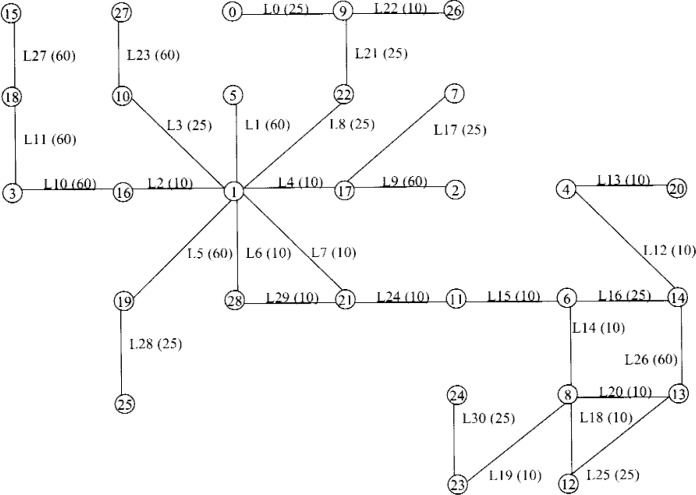
Test Set DATA13.

**Fig. A24:**
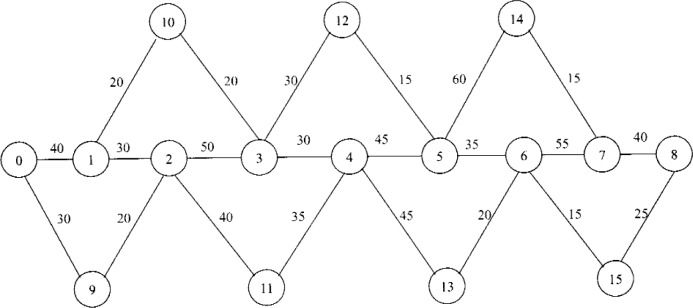
Test Set DATA14.

## 6. Appendix A. BWP Test Sets

These network test sets are those referred to in Tables 2 through 4 in Sec. 4.
